# Genetic Variability in Beta-Defensins Is Not Associated with Susceptibility to *Staphylococcus aureus* Bacteremia

**DOI:** 10.1371/journal.pone.0032315

**Published:** 2012-02-22

**Authors:** Peder Fode, Anders Rhod Larsen, Bjarke Feenstra, Cathrine Jespersgaard, Robert Leo Skov, Marc Stegger, Vance G. Fowler, the Danish SAB Study Group Consortium, Paal Skytt Andersen

**Affiliations:** 1 Department for Microbiological Surveillance and Research, Statens Serum Institut, Copenhagen, Denmark; 2 Department of Epidemiological Research, Statens Serum Institut, Copenhagen, Denmark; 3 Department of Clinical Biochemistry and Immunology, Statens Serum Institut, Copenhagen, Denmark; 4 Department of Infectious Diseases, Duke Medical Center, Durham, North Carolina, United States of America; National Institutes of Health, United States of America

## Abstract

**Introduction:**

Human beta-defensins are key components of human innate immunity to a variety of pathogens, including *Staphylococcus aureus*. The aim of the present study was to investigate a potential association between gene variations in *DEFB1* and *DEFB103/DEFB4* and the development of *S. aureus* bacteremia (SAB) employing a case-control design.

**Methods:**

Cases were unique patients with documented SAB, identified with the National *S. aureus* Bacteremia Register, a comprehensive dataset of all episodes of community associated-SABs (CA-SAB) occurring in children (≤20 yrs) in Denmark from 1990 to 2006. Controls were age-matched healthy individuals with no history of SAB. DNA obtained from cases and controls using the Danish Newborn Screening Biobank were genotyped for functional polymorphisms of *DEFB1* by Sanger sequencing and copy number variation of the *DEFB103* and *DEFB4* genes using Pyrosequencing-based Paralogue Ratio Test (P-PRT).

**Results:**

193 ethnic Danish SAB cases with 382 age-matched controls were used for this study. *S. aureus* isolates represented a variety of bacterial (i.e., different *spa* types) types similar to SAB isolates in general. *DEFB1* minor allele frequencies of rs11362 (cases vs. controls 0.47/0.44), rs1800972 (0.21/0.24), and rs1799946 (0.32/0.33) were not significantly different in cases compared with controls. Also, *DEFB4/DEFB103* gene copy numbers (means 4.83/4.92) were not significantly different in cases compared with controls.

**Conclusions:**

Using a large, unique cohort of pediatric CA-SAB, we found no significant association between *DEFB1* genetic variation or *DEFB4/DEFB103* gene copy number and susceptibility for SAB.

## Introduction

*Staphylococcus aureus* is a leading cause of bacteremia and endocarditis in the industrialized world. In 2008, the incidence of *S. aureus* bacteremia (SAB) in Denmark was 25.6 cases per 100,000 inhabitants [1]. Approximately 20% of these cases had a fatal outcome [1].

A growing body of evidence indicates that host genetic factors are involved in susceptibility to a variety of bacterial pathogens, including meningococci [2], *Mycobacterium tuberculosis* [3], and leprosy [4], [5]. Similar evidence also exists for genetic susceptibility to *S. aureus*. For example, higher rates of invasive *S. aureus* infection have been described in ethnically distinct populations, including Australian Aborigines, New Zealand Maori, and Canadian Native Americans [6]. Using a murine *S. aureus* sepsis model, Ahn *et al.* recently demonstrated that inbred A/J mice were highly susceptible to *S. aureus* infection as compared to C57BL6, and that this susceptibility was associated with regions on A/J chromosomes 8, 11, and 18, chromosomes that included defensin genes and several other innate immune genes [7]. Additionally, investigators at Erasmus University recently suggested that variability within key innate immunity genes were associated with persistent *S. aureus* carriage [8]. Despite these findings, however, the precise genetic determinants for susceptibility to *S. aureus* infection in humans are unknown.

Defensins are small cationic peptides with antimicrobial activity [9]–[12] and shown to be important players in the innate immune system. The majority of the defensin genes including *DEFB1*, *DEFB4* and *DEFB103* encoding human beta-defensins 1, 2 and −3, respectively [13], are located on chromosome 8p23.1, a region of great complexity with both functional SNPs (*DEFB1*) and copy number variation (CNV) of a wide range of beta-defensin genes including *DEFB4* and *DEFB103*. *DEFB1* is apparently constitutively expressed [14], but functional single nucleotide polymorphisms (SNPs) in *DEFB1* have been associated with susceptibility to severe sepsis [15]. Furthermore, human beta-defensin 3 (HBD3) has a strong activity against *S. aureus* [16]. The genomic copy number (GCN) for *DEFB4/DEFB103* varies from 2 to 12 copies per diploid genome [13]. Interestingly, an increased GCN of *DEFB4/DEFB103* is associated with exacerbations of psoriasis, an inflammatory skin disease characterized by a notable absence of *S. aureus* colonization [17].

The combination of strong activity against *S. aureus*, functional *DEFB1* SNPs and high variability in *DEFB4/DEFB103* GCN makes these genes promising candidates for human susceptibility to *S. aureus* infections. In the present study we used a unique cohort of all pediatric patients with SAB in Denmark over a 16-year period to evaluate potential associations between either functional SNPs of *DEFB1* or low GCN of *DEFB4/DEFB103* and pediatric SAB.

## Materials and Methods

### Ethics Statement

The present study was approved by the Regional Science Ethics Committee of Copenhagen (2007-0104), the Danish Data Protection Agency (2008-54-0458), and The Danish Newborn Screening Biobank's review board a similar, but more independent review board than an Institutional Review Board. The parents are informed verbally and by homepage (www.ssi.dk/nyfoedte) about the use of The Danish Newborn Screening Biobank and can opt out at any time [18].

### Identification of cases and controls

The present study included all Danish SAB pediatric patients in the period 1990–2006. For the purposes of this study, pediatric patients were defined as ≤20 years of age at the time of their SAB episode. SAB acquisition was classified as Hospital associated (HA)-SAB, Community associated (CA)-SAB or unknown, based on the time of positive SAB sampling after hospital admission. A SAB episode was registered as CA-SAB if diagnosed in less than 48 h after hospital admission based on information retrieved prospectively from discharge summaries. These data were retrieved from The Danish *S. aureus* Bacteremia Database at SSI, containing all Danish SAB isolates and corresponding bacteriological and patient data since 1958. Patients were included if they were born after 1982, were of Danish origin, and had CA-SAB. Danish origin was defined as individuals where both of the individual's parents were born in Denmark.

For each case two Danish individuals (same definition as for cases) from The Danish Newborn Screening Biobank who according to the Danish National Patient Registry had not been diagnosed with SAB or other severe bacterial infections served as controls. Samples for these control samples were adjacent to the case samples in the biobank regardless of gender. Two controls suffered from diabetes and two had died from cancer.

### Bacteriological data

All *S. aureus* isolates were retrieved from The Danish *S. aureus* Bacteremia Repository and were *spa*-typed according to standard practise and assigned to clonal complexes (CC) [19].

### DNA extraction

DNA was extracted from 3 mm filter paper blood samples using Generation DNA Elution solution and DNA purification solution (Qiagen, Valencia, CA) as described by Baker *et al.* [20]. Extraction was performed in 96-well format with each well containing a dried blood sample. Washing buffer (PBS, 0.5% Tween20) was added to each well and the samples were shaken at 1000 rpm for 45 min at room temperature before the supernatant was removed. The washing procedure was repeated. Purification solution was added and the supernatant was removed. The procedure was repeated. An elution solution was then added and after 5 min the supernatant was removed. Sterile water was added, and the plate was placed at −20°C for 15 min before being heated to 99°C for 15 min.

### *DEFB1* SNP analysis

Genotyping of three *DEFB1* 5′UTR SNPs (rs11362 [−20G/A]; rs1800972 [−44 C/G] and rs1799946 [−52 G/A]) was performed using Sanger DNA sequencing after obtaining a 318 bp fragment (Forward primer: 5′-CTC CCT TCA GTT CCG T-3′ and reverse primer 5′-CTT GTT CCT CGT CCC TT-3′). Hardy-Weinberg equilibrium was seen in cases and controls separately and combined for all three SNPs.

### *DEFB4/DEFB103* Copy number determination

Gene copy number was determined using the Pyrosequencing-based Paralogue Ratio Test (P-PRT). The method is based on the Paralogue Ratio Test (PRT) described previously [21], [22]. In brief, the *DEFB103* region on chromosome 8 and an identified paralogue gene (*HSPD21* on chromosome 21) with only two copies per genome were PCR amplified using one set of primers. The resulting PCR amplicons differed at 10 positions. One of the positions where the amplicons differed was used to quantify the two chromosome regions against each other by pyrosequencing across it. The P-PRT method used has recently been described [23].

Primers for the pyrosequencing assay were designed using the PSQ assay design software version 1.0.6 (Qiagen, Hilden, Germany). The following sequence was analysed with the position that varied between chromosomes 8 and 21 marked in bold with underline: KATGC**Y**AT. For the PCR, 20 ng of template DNA in a total volume of 50 µl using a forward primer (5′-GAGGTCACTGTGATCAAAGAT-3′) and a reverse primer (5′-Biotin- AACCTTCAGCACAGCTACTC-3′) was used. Pyrosequencing was carried out using 40 µL of the PCR product and a sequencing primer (5′-AGGTCACTGTGATCAAAGAT-3′) on the PSQ 96 MA Pyrosequencer according to manufacturer's recommendations (Qiagen).

The relative percentages of the two variants were calculated by the Pyrosequencing software (Qiagen) and used for the gene copy number determination. Positive controls from Coriell Cell Repositories with known copy number were included in each run (NA07048: 4 copies; NA10846: 5 copies; NA10847: 7 copies and NA10861 3 copies) and used to generate a correction curve by linear regression. Corrected copy number estimates were calculated for each sample using this run-specific regression equation. A “No template control” was included in each run.

### Statistical analysis

In the *DEFB1* SNP analyses, we tested for association between SNP genotype and case-control status using an additive genetic model. We also tested for possible non-additive effects using dominant, recessive, and full genotype models. Association tests were performed using PLINK [24]. Mean gene copy numbers of cases and controls were compared using unpaired t-test with the Welch correction. If one of the groups did not have a Gaussian distribution, a Mann–Whitney test was performed. Copy number analyses were performed using R (http://www.r-project.org).

### Power calculations

**Figure 1:**
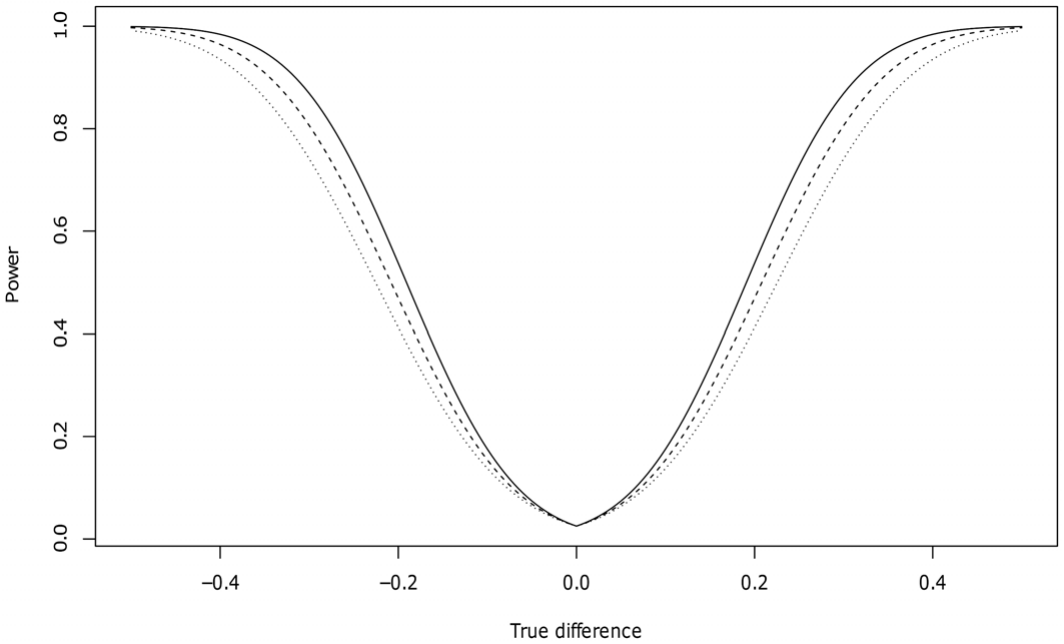
Power curves for an unpaired t-test with 193 cases and 382 controls, assuming equal variance in cases and controls and a significance level of 0.05. True difference in mean copy number is shown on the x-axis, and the power to detect such differences is shown on the y-axis. Curves are shown for three different copy number standard deviations: 1.1 (solid), 1.2 (dashed), and 1.3 (dotted).

In the design phase of the study, we used published association results between higher copy number for β–defensin genes and risk of psoriasis [25] to estimate power for various sample sizes. The effect sizes seen in the study by Hollox et al [25] ranged from 0.25 to 0.4 in mean copy number difference between cases and controls, and the standard deviations of copy numbers in cases and controls ranged between 1.1 and 1.3. Using an unpaired t-test with an significance level of α = 0.05 and assuming equal variances in cases and controls, the power estimates based on these effect sizes ranged from 0.58 (true difference 0.25, standard deviation 1.3) to 0.98 (true difference 0.4, standard deviation 1.1) for 193 cases and 382 controls (see Figure 1 for power curves). Using the largest observed standard deviations in our study (1.17 in cases), the power was 0.67 for a true effect size of 0.25, and for an effect size of 0.40 the power was 0.97. Power calculations were performed using R.

## Results

A total of 1,674 patients ≤20 years were diagnosed with SAB in Denmark from 1990 to 2006. Of these patients, 464 had CA-SAB, 882 had HA-SAB, and for 328 patients the origin of infection remained unknown. Among the CA-SAB pediatric cases, 193 patients met inclusion criteria being born after 1982 and had an available blood sample in The Danish Newborn Screening Biobank for DNA extraction. These 193 patients constitute the cases for the present study.

Next, we identified control subjects. A total of two controls per sample were selected for the study, but four had to be excluded due to lack of DNA in the samples resulting in 382 controls. The cases included 130 males (67%) and 63 females (33%). Among the controls 229 (60%) were males and 153 (40%) were females. There was no significant difference in gender distribution between cases and controls (χ^2^ = 2.69, *P* = 0.10). The gender distribution among the included cases and controls was also similar to HA-SAB in patients ≤20 years of age and to SAB patients in general.

### Distribution of *spa* types and antibiogram

All isolates were susceptible to methicilin. The diversity of the *S. aureus* isolates was determined by *spa* typing to elucidate if the distribution was biased, e.g. whether specific types of *S. aureus* were overrepresented in CA-SAB. We did, however, not find any such bias as the distribution of phage groups were similar in both the selected cases and other SAB patient cases both within the same period and age group as well as among the total number of cases retrieved from the SAB register (Table 1). A total of 113 different *spa* types were found among 192 isolates that were assigned to 18 known CC groups. The remaining 31 isolates were either missing (n = 7) or had atypical *spa* repeats that could not be assigned. The most prevalent CC groups were: CC45 (26.3%), CC30 (20.4%), CC15 (12.9%), CC509 (6.5%), CC121 (6.5%), and CC8 (4.8%).

**Table 1:** Phage distribution among SAB cases 1990–2006.

*Phage pattern*	*Phage type*	*CA-SAB pts.≤20 yrs.*	*Other SAB≤20 yrs.*	*All SAB*
		*No. (%)*	*No. (%)*	*No. (%)*
80 Complex	80, 81+combination of 52 and/or 52A with 80 and/or 81	3 (1.6)	27 (1.6)	1547 (6.4)
Rest of phage group I	29, 52, 52A, 79, 80	30 (15.5)	248 (14.8)	4396 (18.1)
Phage group II	3A, 3C, 55, 71	39 (20.2)	357 (21.4)	4256 (17.6)
Phage group III	6, 42E, 47, 53, 54, 75, 77, (81), 83A, 84, 85, 89, 93	23 (11.9)	201 (12.0)	3561 (14.7)
83A Complex	Combination of one or more of only 83A, 84, 85, 89, 93	9 (4.7)	74 (4.4)	966 (4.0)
94,96 Complex	94, 96	3 (1.6)	73 (4.4)	1596 (6.6)
Type 95	95	25 (13.0)	317 (18.9)	3864 (16.0)
NI (mixed phage group)	Mixture of the reactions in the phage type pattern above	28 (14.5)	155 (9.3)	1695 (7.0)
NT(non typable	Non typable at phage concentration 1000× Routine Test Dilution (RTD)	33 (17.1)	222 (13.3)	2347 (9.7)

### *DEFB1* SNP analysis

Patients and controls were genotyped for three *DEFB1* promoter SNPs, rs11362 [−20G/A]; rs1800972 [−44 C/G] and rs1799946 [−52 G/A]. Results of the genotyping are given in Table 2. The minor allele frequencies of the three SNPs did not differ between SAB cases and controls (*P*>0.05 for all three SNPs). Analyses with dominant, recessive and full genotype models as well as haplotype analyses also did not show any differences between cases and controls (data not shown).

**Table 2:** *DEFB1* Genotype and minor allele frequencies in CA-SAB cases and controls.

	CA-SAB	Controls	*P* value
	*n* (%)	*n* (%)	
[−20G/A] (rs11362)			
GG	60 (31)	123 (32)	
GA	85 (44)	182 (48)	
AA	48 (25)	76 (20)	
Minor allele (A)	181(47)	334 (44)	*P* = 0.32
[−44 C/G] (rs1800972)			
CC	119 (62)	224 (59)	
CG	68 (35)	133 (35)	
GG	7 (4)	23 (6)	
Minor allele (G)	82 (21)	179 (24)	*P* = 0.36
[−52 G/A] (rs1799946)			
GG	93 (48)	170 (45)	
GA	78 (40)	171 (45)	
AA	23 (12)	39 (10)	
Minor allele (A)	124 (32)	249 (33)	*P* = 0.78

### *DEFB4/DEFB103* Copy number determination

**Figure 2:**
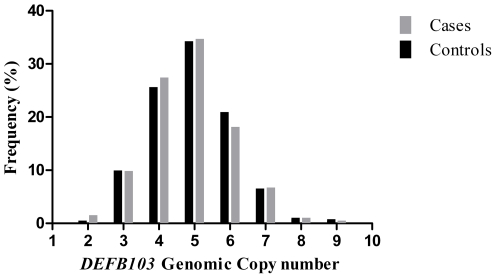
Frequency distribution of *DEFB4/DEFB103* GCN for SAB cases and controls.

Gene copy number for *DEFB4/DEFB103* varied from 2 to 9 in both CA-SAB cases and controls with comparable frequency distributions for the two groups (Figure 2). Mean copy numbers (and standard deviations) for cases and controls were 4.83 (1.17) and 4.92 (1.12). There was no significant difference in copy number between cases and controls (*P* = 0.33, t-test). However, a Shapiro-Wilks test showed deviation from normality in the copy number distribution for the controls (*P* = 0.002). We therefore also applied a Mann-Whitney test, but again we found no significant difference in copy number between cases and controls (*P = 0.37*).

## Discussion

Using a large, Caucasian Danish, and clinically well-described bacteremia registry of children with CA-SAB, we found no association between the *DEFB1* SNPs (rs11362 [−20G/A]; rs1800972 [−44 C/G] and rs1799946 [−52 G/A]) or the *DEFB4/DEFB103* GCN and susceptibility to CA-SAB [23]. The CA-SAB isolates in our registry were of diverse types indicating that no specific type was dominant among CA-SABs.

We have established a large well-characterized nested CA-SAB case-control study (193 cases and 382 controls), which has allowed us to study genetic association in an ethnic homogenous population. This is among the largest CA-SAB cohorts for genetic association studies, and our study had good statistical power to detect case-control differences in mean copy number larger than 0.25. It is, however, possible that weaker genetic associations exist that will not be detected with a cohort of this size. The GCN was determined by P-PRT, which we previously have shown to be a reliable method [23], but like other GCN assays is associated with errors. However, in the present study the relative differences between cases and controls rather than exact GCN are the principal aim where we assume similar error rates in cases and controls.

Defensin genes have been the focus of the present susceptibility study as the functional consequences of such mutations appear to be associated with reduced expression and presumably reduced activity of the resulting peptides. A promoter polymorphism (rs11362) of *DEFB1* has previously been shown to be associated with sepsis [15]. However, we did not see any association between either the *DEFB1* polymorphism or other closely positioned polymorphisms and SAB in a study population comparable in size (193 vs. 211) to that of Chen and co-workers [15]. The genetic association previously found could thus be the result of infection by other types of bacteria than SAB or the difference could be due to ethnic differences between the two studies or the fact that our study is focused only on *S. aureus* bacteremia cases in contrast to the broader study of Chen and co-workers who did not distinguish between different bacterial infections.

It is intriguing that we now have the possibility to address the issue of genetic association to a specific type of bacterial infection. Addressing other candidate genes based on information from murine studies and *in silico* mapping [7] may give further insight in the genetic susceptibility to *S. aureus* bacteremia.
